# Combination of suberoylanilide hydroxamic acid with heavy ion therapy shows promising effects in infantile sarcoma cell lines

**DOI:** 10.1186/1748-717X-6-119

**Published:** 2011-09-20

**Authors:** Susanne Oertel, Markus Thiemann, Karsten Richter, Klaus-J Weber, Peter E Huber, Ramon Lopez Perez, Stephan Brons, Marc Bischof, Andreas E Kulozik, Volker Ehemann, Jürgen Debus, Claudia Blattmann

**Affiliations:** 1 Department of Radiooncology, University of Heidelberg, (INF 400), Heidelberg, (69120), Germany; 2 Core Facility Electron Microscopy, German Cancer Research Center, (INF 280), Heidelberg, (69120), Germany; 3 Department of Radiation Oncology, German Cancer Research Center, (INF 280), Heidelberg, (69120), Germany; 4 Heidelberger Ionentherapiezentrum (HIT), (INF 672), Heidelberg, (69120), Germany; 5 Department of Pediatric Oncology, Hematology and Immunology, University Children's Hospital, (INF 672), Heidelberg, (69120), Germany; 6 Institute of Pathology, University of Heidelberg, (INF 220), Heidelberg, (69120), Germany

**Keywords:** Infantile sarcoma, histone deacetylase inhibition, heavy ion radiotherapy, suberoylanilide hydroxamic acid, SAHA

## Abstract

**Introduction:**

The pan-HDAC inhibitor (HDACI) suberoylanilide hydroxamic acid (SAHA) has previously shown to be a radio-sensitizer to conventional photon radiotherapy (XRT) in pediatric sarcoma cell lines. Here, we investigate its effect on the response of two sarcoma cell lines and a normal tissue cell line to heavy ion irradiation (HIT).

**Materials and methods:**

Clonogenic assays after different doses of heavy ions were performed. DNA damage and repair were evaluated by measuring γH2AX via flow-cytometry. Apoptosis and cell cycle analysis were also measured via flow cytometry. Protein expression of repair proteins, p53 and p21 were measured using immunoblot analysis. Changes of nuclear architecture after treatment with SAHA and HIT were observed in one of the sarcoma cell lines via light microscopy after staining towards chromatin and γH2AX.

**Results:**

Corresponding with previously reported photon data, SAHA lead to an increase of sensitivity to heavy ions along with an increase of DSB and apoptosis in the two sarcoma cell lines. In contrast, in the osteoblast cell line (hFOB 1.19), the combination of SAHA and HIT showed a significant radio-protective effect. Laser scanning microscopy revealed no significant morphologic changes after HIT compared to the combined treatment with SAHA. Immunoblot analysis revealed no significant up or down regulation of p53. However, p21 was significantly increased by SAHA and combination treatment as compared to HIT only in the two sarcoma cell lines - again in contrast to the osteoblast cell line. Changes in the repair kinetics of DSB p53-independent apoptosis with p21 involvement may be part of the underlying mechanisms for radio-sensitization by SAHA.

**Conclusion:**

Our *in vitro *data suggest an increase of the therapeutic ratio by the combination of SAHA with HIT in infantile sarcoma cell lines.

## Introduction

HDAC inhibitors (HDACI) induce growth arrest and affect cell differentiation, apoptosis and anti-angiogenic effects in tumor cells by chromatin modification with both transcription-dependent and independent mechanisms implicated [1,2].

Suberoylanilide hydroxamic acid (SAHA) is the first HDACI that has been approved in the United States by the Food and Drug Administration (FDA) for the treatment of relapsed and refractory cutaneous T-cell lymphoma. It has also shown promising preclinical results *in vitro *and *in vivo *for several other cancer types [3-5]. Interesting selective synergistic effects by combination of SAHA with other cytotoxic agents, amongst others radiation, have been reported for osteosarcoma cells [6,7] as well as for many other types of cancer cells [8-10].

In a previous report, we have shown that SAHA enhances radio-sensitivity to conventional megavoltage photon beam radiation (XRT) in multiple pediatric sarcoma cell lines [7].

DNA double-strand breaks (DSBs) arise from exposure to ionizing radiation. Cells have evolved mechanisms to repair these lesions that are otherwise lethal. These mechanisms involve phosphorylation of histone H2AX (then called γH2AX) and the loading of repair proteins on the chromatin adjacent to the DSBs. It has also been shown that the chromatin architecture in the region surrounding the DSB has a critical impact on the ability of cells to mount an effective DNA damage response [11].

As SAHA is known to modify chromatin structure, we investigated the changes in γH2AX-expression after irradiation and were able to find a correlation of increased radiosensitivity with increased γH2AX-expression as well as prolongation of radiation-induced γH2AX-expression in the sarcoma cell lines, but interestingly not in normal tissue cell lines when SAHA was combined with XRT [C. Blattmann, submitted]. As DSBs are known to occur with a higher frequency in response to heavy ions compared to photon irradiation [12] we now were interested in the combination of heavy ion radiation with HDACIs.

Heavy ion therapy (HIT) with carbon ions has achieved superior cancer control in tumors with otherwise low radiosensitivity, like sarcomas [13]. Several evident as well as potential advantages over XRT have lead to a wider popularization of HIT with a number of new facilities that have become operational worldwide. First *in vitro *data show promising effects by the combination of HIT and SAHA in esophageal cancer cells [14].

Here we investigate the effect of the HDACI SAHA in combination with HIT on two pediatric sarcoma cell lines (KHOS24-OS (osteosarcoma), A-204 (rhabdomyosarcoma)), as well as a normal tissue cell line (HFOB1.19, human osteoblast).

## Materials and methods

### Cell lines

Human sarcoma cell lines (KHOS24-OS and A-204), as well as the human osteoblast hFOB 1.19 were obtained from the American Type Culture Collection (ATCC; Rockville, MD).

### Chemicals

SAHA was obtained from Alexis Biochemicals (Lörrach, Germany). Primary monoclonal mouse antibodies against Rad51, Ku70 and Ku80, p21 and p53 were obtained from Abcam (Cambridge, UK). Primary monoclonal mouse antibodies against ß-actin as well as a secondary antibody for immunoblot experiments were purchased from CellSignaling Technology (Danvers, MA, USA). For the flow cytometry experiments as well as immunoblots, γH2AX antibody Alexa Fluor^® ^488 anti-H2A.X-phosphorylated (Ser139) was obtained from BioLegend (San Diego, USA).

### Clonogenic assay

Clonogenic assays were performed as described previously [7]. In brief, exponentially growing tumor cells were plated in T25 culture bottles at appropriate numbers to give an estimated 50-250 colonies/flask and were incubated with medium containing 0 to 5 μM SAHA. Incubation of SAHA with the respective LD_20 _and LD_50 _for each cell line started 24 h before XRT/HIT. Incubation was stopped after 5 days. Monolayers were stained with 0.5% crystal violet for 10 minutes. Plates were stained with 0.1 M sodium citrate (pH 4) in ethanol 100% (3:1) for another 10 minutes. Afterwards, plates were dried for 48 to 72 h and colonies were counted manually. Survival was defined as the ability of cells to form colonies (≥ 50 cells).

Surviving fractions were obtained by normalizing the plating efficiencies (cell number/plated cell) to the respective control values. Each experiment was done in triplicate and at least three independent repetitions were performed. In combination experiments, the survival rates after different doses of radiation were normalized to the treatment with SAHA given alone.

Following a theoretical concept of combination effects by Steel and Peckham [15,16] the range of additivity was calculated from the response to the LD 20 of the single agent. This range is encompassed by the prediction of independent cell killing (accounted for by normalizing the radiation survival rates to SAHA toxicity, see above) and a theoretical survival curve that can be obtained if the fraction of cells surviving drug treatment is formally treated as being irradiated with an isoeffective dose *D*¢. Assuming that the radiation sensitivity coefficients were ax and bx, one readily finds that the theoretical survival curve (normalized to drug toxicity) can be written as SF = exp(- a p *D*- bx*D*2) with a p = ax + 2bx*D*¢. For graphical representation of the combination effect in excess of independent cell killing, both the experimental survival fraction (cell number/plated cells) and the fitted survival curves were multiplied with the averaged surviving fraction after SAHA exposure alone.

### Flow cytometric analysis of γH2AX-expression, cell cycle and apoptosis

Cells were seeded in T25 culture plates at a density of 1 × 10^6 ^cells per plate 24 h before HIT. In the SAHA experiments, 0.5-1 μM SAHA was added 24 h before HIT. At certain time points after HIT, cells were harvested and centrifuged (800 g). Cells were washed with PBS several times and then fixed with 3% paraformaldehyde (PFA, Sigma) for 10 min at room temperature (RT). Ice-cold methanol (90%) was added and samples were kept on ice for another 30 min. Afterwards, samples were washed three times in 0.5% BSA/PBS re-suspended in 100 μl 0.5% BSA/PBS and incubated for 10 min at room temperature. Cells were stained for γH2AX by 1 h incubation at RT in 10 μl antibody plus 90 μl 0.5% BSA/PBS per sample. Finally, cells were washed three times with 0.5% BSA/PBS. Cells were further stained with DAPI for cell cycle analysis for 30 min at RT and analyzed simultaneously with the γH2AX staining. The samples were analyzed directly on a "Galaxy Pro"- Flowcytometer from Partec (Münster, Germany). The relative fluorescence intensity in the gated areas was calculated using the multiparameter "Flow max" software from Partec as described in a further report. For the detection of apoptotic cells we used Nicoletti stain measured in a FACS Calibur Flow cytometer (Becton Dickinson Cytometry Systems, San Jose, CA) as described in our earlier report [7].

To assess the mean extent of DNA damage at a particular phase of the cell cycle, the mean values of γH2AX immunfluorescence were calculated separately for G_0/1_, S and G_2_/M cells by the computer-interactive "gating" analysis. Cells in S and G_2_/M have 1.5 and 2.0 times higher γH2AX mean immunofluorescence respectively, compared to cells in G_0/1 _because of the increase of DNA and histone content during the cell cycle. Therefore, the data has to be normalized for DNA (histone) content by dividing the mean γH2AX immunofluorescence of S- and G_2_/M-phase cells by 1.5 and 2.0, respectively. Finally, a low level of γH2AX immunofluorescence is seen in the untreated cells which represent an "intrinsic" γH2AX phosphorylation. Therefore, the γH2AX immunofluorescence level of the untreated controls has to be subtracted from the immunofluorescence level of the treated cells in order to get the γH2AX immunofluorescence level which is treatment-related. Then multiparametric analysis was done on a Galaxy pro flow cytometer (PARTEC, Münster, Germany) by stimulating the fluorochromes DAPI with mercury 100 W vapour lamp, H2AX-FITC with a 488 nm air cooled argon laser and measuring the fluorescence intensities at 530/30 nm and apoptotic cells stained with Nicoletti stain/DAPI/propidium iodide (PI) at 610/20 nm. The green fluorescent FITC, and red fluorescent PI, was measured in the logarithmic mode, DAPI stained DNA measured in linear mode. Analysis and calculation the Flow Max software (Partec, Münster, Germany was used. Each analysis represents 5000 cells.

### Immunoblot analysis

Immunoblot analysis was performed as reported previously [7]. In brief, following treatment, cells were lysed with lysis buffer (0.5 M tris/Cl, pH 6,8, SDS, 87% Glycerin, DTT 1 M ad Aqua 100 ml). Following this, 1 ml lysate was incubated with 1 μl benzonase for 15 min at 37°C. 40 μg of protein extracts underwent electrophoresis onto a 12% polyacrylamide gel (Pierce Biotechnology Inc., Roxford, IL, USA) under reducing conditions. The separated proteins were transferred onto nitrocellulose membranes (Amersham Pharmacia Bioscience, Piscataway, NJ). The membranes were then incubated for 45 minutes in blocking buffer (tris-buffered saline with 0.1% Tween (TBS-T) and 5% nonfat dry milk), followed by incubation with specific primary antibodies at 1:1000 dilution at -4°C for 24 h or at room temperature for 1 h. After being washed with TBS-T buffer three times, the membrane was incubated with anti-mouse IgG secondary antibody (Cell Signaling Technology, Danvers, MA, USA) at 1:1000 dilution for 1 h at room temperature. The signals were visualized with the ECL+ detection system and autoradiography.

### Confocal Laser Scanning Microscopy

The nuclear organization of chromatin and γHSAX in KHOS-24 OS cells upon treatment with SAHA, HIT and both in combination was observed by laser scanning microscopy using a Zeiss LSM 700, equipped with a 63 × oil objective. Images were acquired with pinhole-size 1 Airy at pixel-size 100 nm. Cells were fixed with buffered 2% formaldehyde, chromatin stained with DAPI and γH2AX revealed by IHC using Alexa 488-charged secondary antibody as reporter.

### Statistical analysis

All experiments were performed at least twice. Furthermore, each experiment was done in duplicate. Clonogenic assays were performed in triplicate. Combination studies were evaluated using student's *t *test with the resulting *p *value representing a two-sided test of statistical significance.

## Results

**Figure 1:**
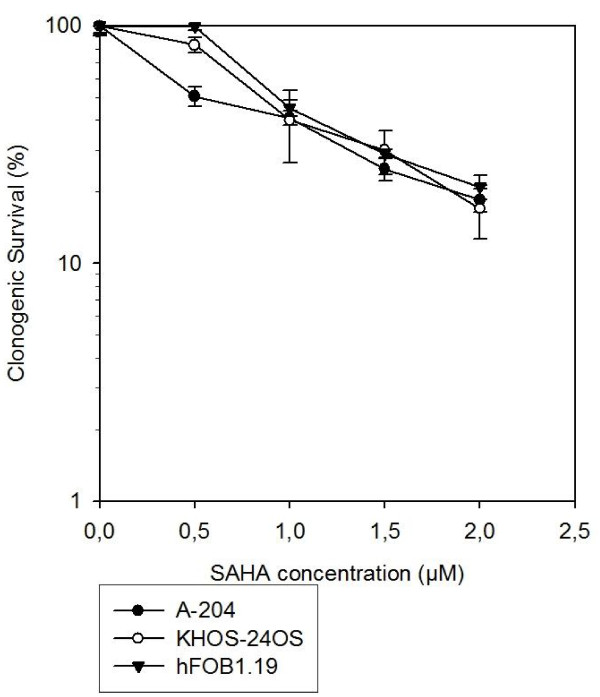
**Clonogenic Survival after SAHA Treatment**. Suberoylanilide hydroxamic acid (SAHA) decreases clonogenic survival in pediatric sarcoma cell lines (KHOS-24OS, A-204) as well as an osteoblast cell line (hFOB 1.19) in a dose-dependent manner.

We determined the survival of the two sarcoma cell lines (KHOS-24OS and A-204) as well as a human cell line (hfOB 1.19) exposed to combination therapy of SAHA and HIT using clonogenic assays. The cell lines were pretreated with 0.25 and 0.5 μM SAHA for A-204, and 0.5 and 1 μM SAHA for KHOS-24OS as well as hFOB1.19 24 h before HIT. The SAHA concentrations correspond well to clinically achievable plasma concentrations shown in phase I studies of SAHA in adult patients [16] and represent the LD_20 _and LD_50 _for the cells as shown in Figure 1.

**Figure 2:**
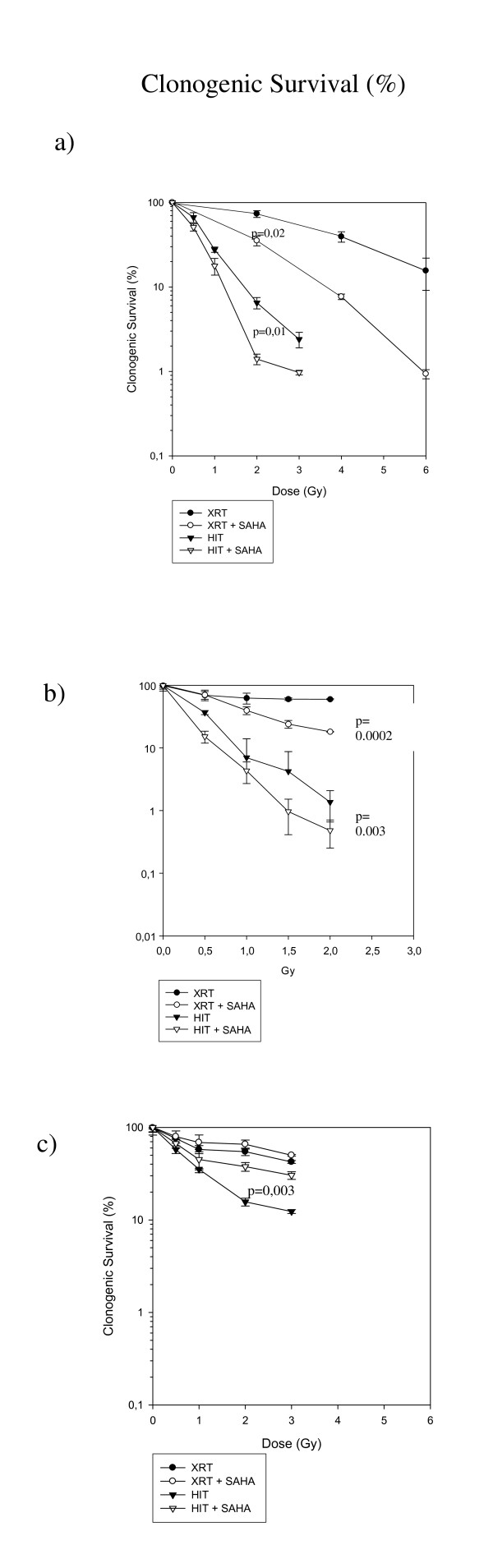
**Clonogenic Survival after Radiation +/- SAHA Treatment at LD**_**50**_. Clonogenic survival of KHOS-24OS (**a**) and A-204 (**b**) and hFOB1.19 (**c**) treated with different doses of conventional photons (XRT) and carbon ions (HIT) with and without the respective LD_50 _dose of SAHA for each cell line (added to the medium 24 h prior to radiation).

**Figure 3:**
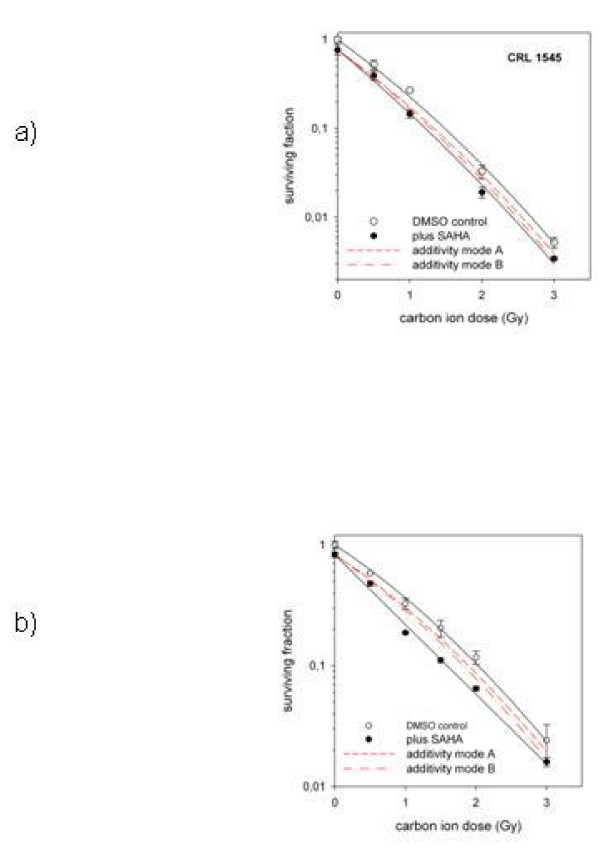
**Survival of sarcoma cells after Radiation +/- SAHA Treatment at LD**_**20**_. Survival of KHOS-24OS and A-204 cells treated with different doses of radiation given alone. (open symbols) or in combination with the LD_20 _of SAHA for each cell line (closed symbols). The data are mean values (and standard deviations) from three independent determinations for each treatment modality. The survival data after combined treatment are normalized to SAHA toxicity alone. The curves represent fits of the linear-quadratic survival expression to the respective data. The slashed lines show the calculated expectation for each modality revealing a supra-additive effect in the real measurements (solid lines).

**Figure 4:**
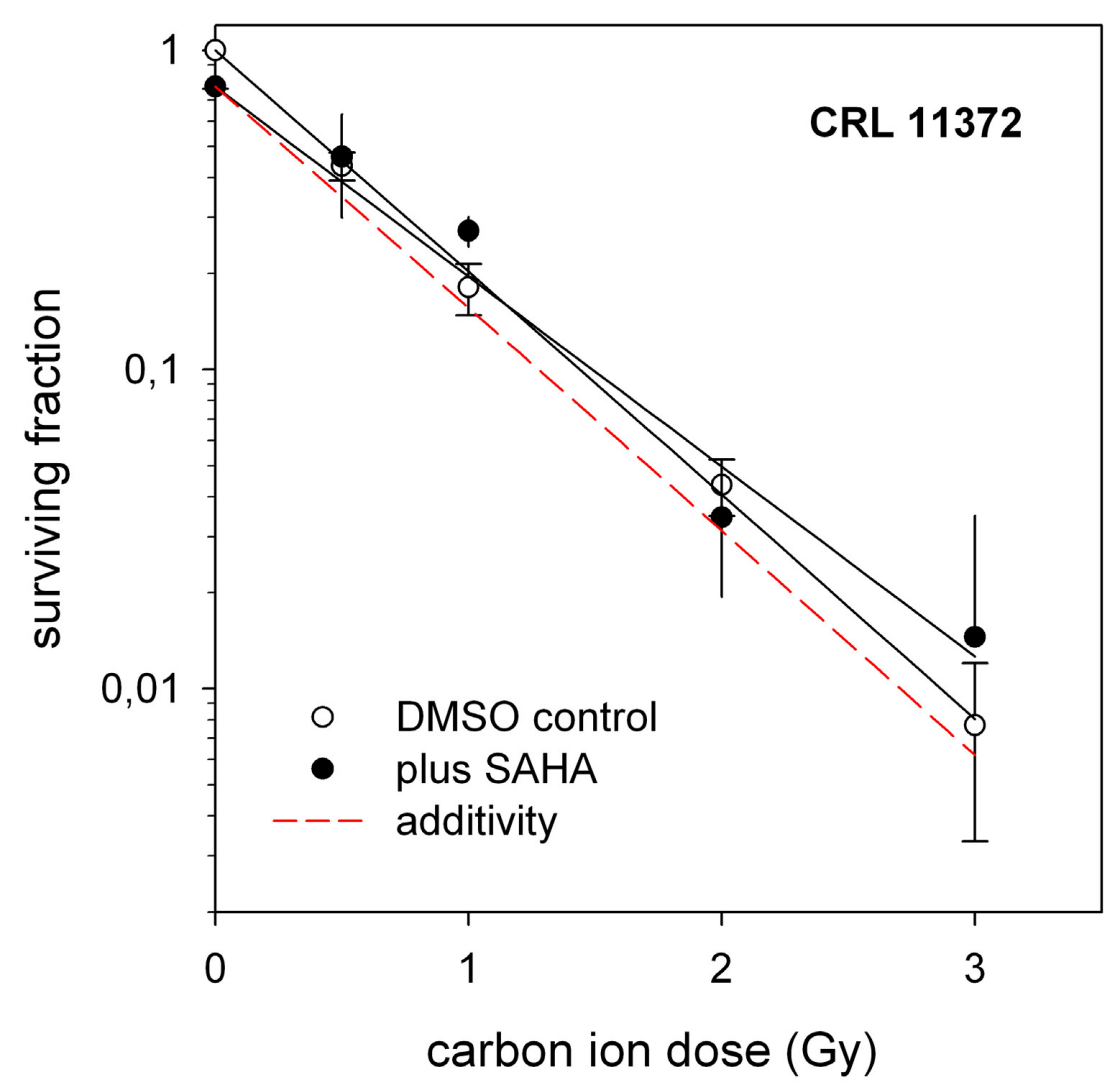
**Survival of Osteoblast Cell line after Radiation +/- SAHA Treatment at LD**_**20**_. Survival of hFOB 1.19 cells treated with different doses of radiation given alone (open symbol) or in combination with the LD_20 _of SAHA (closed symbol). The data are mean values (and standard deviations) from three independent determinations for each treatment modality. The survival data after combined treatment are normalized to SAHA toxicity alone. The curves represent fits of the linear-quadratic survival expression to the respective data. The slashed lines show the calculated expectation for each modality revealing no additivity, but rather a protective effect in the real measurements (solid lines) in hFOB 1.19.

Figure 2 shows that HIT alone suppressed the clonogenic survival significantly more than XRT alone in all three cell lines. The combination with SAHA suppressed the clonogenic survival after radiation with carbon ions significantly more in both pediatric sarcoma cell lines investigated with a stronger effect in KHOS-24OS. The effect of SAHA in combination with heavy ions for KHOS-24OS as well as A.-204 was clearly supra-additive as shown in Figure 3. Interestingly, in contrast SAHA showed a significant radio-protective effect in combination with HIT in the human osteoblast cell line (Figure 4); a tendency we also observed in combination with XRT (not significant) [7].

**Figure 5:**
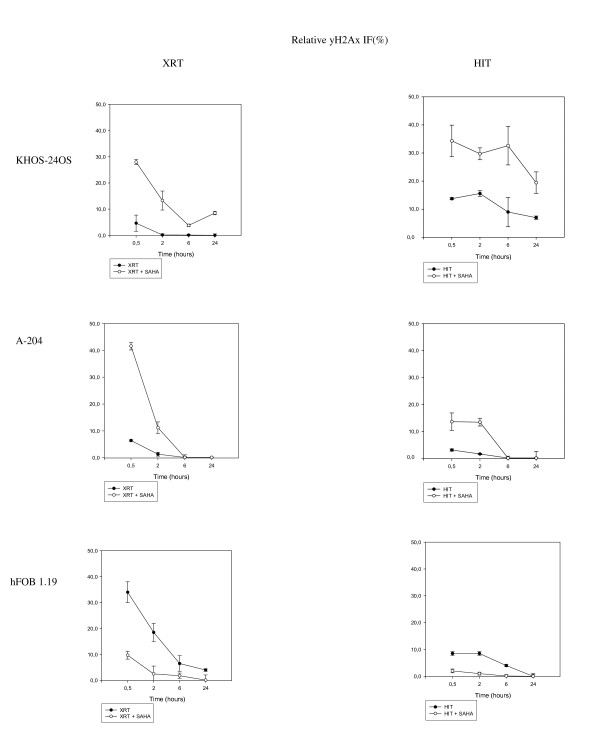
**γH2AX-expression after XRT and HIT**. γH2AX-expression of KHOS-24OS, A-204 and hFOB 30 min, 2 h, 6 h and 24 h after XRT (a) and HIT (b) with and without prior incubation with SAHA as measured by flow cytometry.

We further evaluated γH2AX-expression (Figure 5**) **which has been established as a sensitive indicator of DSB [17], choosing radiation doses of the respective LD80-90 of the sarcoma cell lines. Treatment with SAHA alone had no effect on γH2AX-expression in all cell lines compared to the untreated controls. Just like XRT, HIT resulted in a peak and a following continuous time-dependent drop of mean immunofluorescence (IF) of γH2AX-positive cells in both sarcoma cell lines. Pretreatment with SAHA significantly increased the effect of HIT in KHOS-24OS as well as A-204. In the XRT, but even more in the HIT experiments, the KHOS-24OS cell line showed a prolonged presence of γH2AX, respectively DSB, which was not observed in A-204. In contrast to KHOS-24OS, differences in γH2AX-expression after HIT as well as XRT disappeared 6 h after treatment in the A-204 cell line. With hfOB1.19, corresponding with the results of the clonogenic assays, pretreatment with SAHA reduced the γH2AX induction caused by XRT or HIT, emphasizing the suggested radio-protective effect of SAHA in the normal tissue cell line.

**Figure 6:**
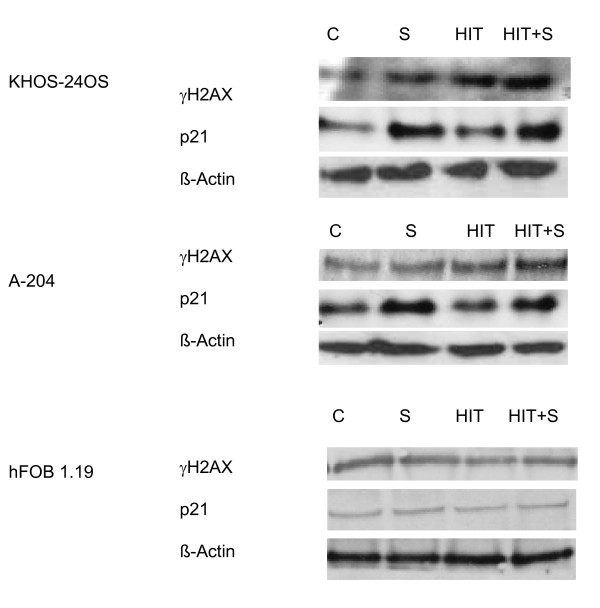
**Immunoblot analyis of γH2AX and p21**. γH2AX-expression measured immunhistochemically 2 hours after HIT and p21 expression in KHOS-24OS, A-204 and HFOB1.19 treated with vehicle control (C), 0,5 (A-204)-1 μM (KHOS-24OS and HFOB1.19) SAHA and HIT (cell specific doses, see Figure 9) or the combination of SAHA and HIT 24 h after HIT.

To confirm our results, we investigated the γH2AX-expression using immunoblot technique. The findings showed that γH2AX-expression was significantly increased 2 hours after HIT or HIT plus SAHA treatment, compared to the untreated cells in KHOS-24OS, as well as A-204, but not in hFOB1.19 (Figure 6).

**Figure 7:**
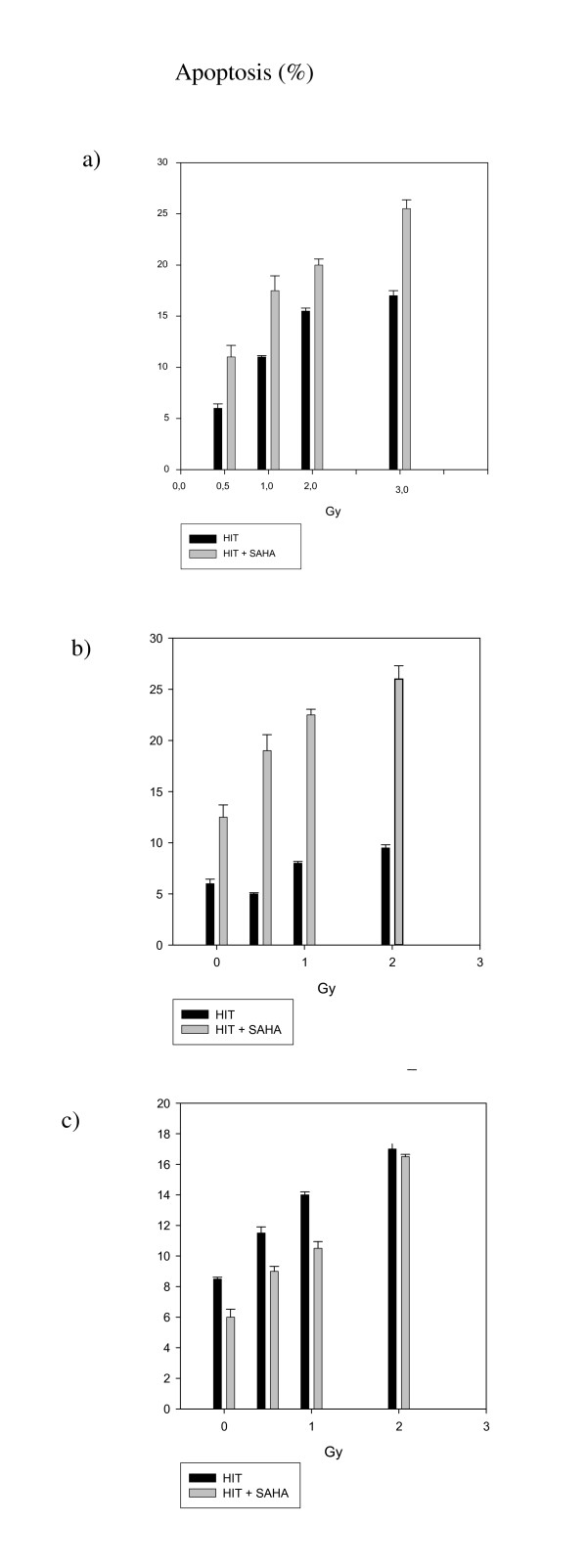
**Apoptosis measured via FACS after HIT +/- SAHA**. Apoptosis of KHOS-24OS (**7**), A-204(**8**) and HFOB1.19(**9**) measured via flow cytometry according to Nicoletti after HIT irradiation with 6 Gy (a), 4 Gy (b) and 4 Gy (c). SAHA was added 24 h before irradiation. Apoptosis was measured 48 h after irradiation.

In our earlier photon experiments we found, that SAHA attenuated key proteins involved in the repair of DSB [7]. Therefore, we again investigated the expression of DNA-DSB repair proteins like Rad51, Ku70 and Ku80 in our HIT experiments. All these proteins play a critical role in the repair of DNA-DSB, and are known to be activated by, amongst others, γH2AX [18]. However, in contrast to our XRT experiments, SAHA did influence expression of either, Rad51, Ku70 and Ku80 measured 24 h after HIT only in the KHOS-24OS, but not in the A-204 cell line when added to the cell culture 24 h before radiation. There was also no change in expression of p53 observable after HIT, SAHA or the combination in all cell lines. However, there were changes in p21 expression. After treatment with SAHA or SAHA plus HIT, p21 was up-regulated in the two sarcoma cell lines, but down-regulated in the osteoblast cell line, compared to the untreated controls and HIT only treatment, respectively (Figure 6). This finding suggests that changes in DNA-repair are less relevant, but that cell cycle regulation changes and a p53-independent apoptotic pathway are the major mechanisms for the sensitization to HIT by SAHA. We further assessed apoptosis 24 and 48 h after HIT. Apoptosis was indeed increased in the KHOS-24OS and A-204 cell line, but rather decreased in the osteoblast cell line 48 h (Figure 7) after irradiation.

**Figure 8:**
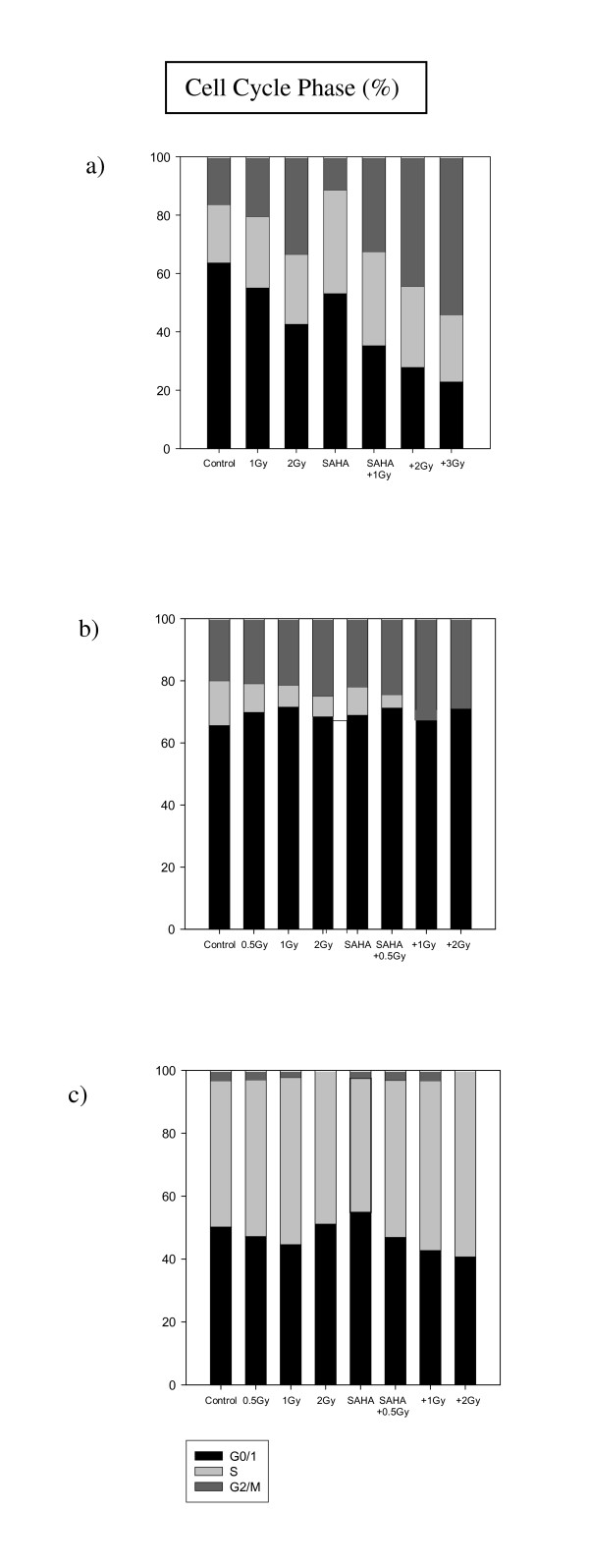
**Cell cycle analysis after HIT +/- SAHA**. Cell cycle analysis in KHOS-24OS (**10**), A-204 (**11**) and HFOB1.19 (**12**) after treatment with HIT +/- SAHA.

Cell cycle observations (Figure 8) of KHOS-24OS revealed a shift into G_0/1 _arrest caused by SAHA, whereas the combination treatment resulted rather in a shift towards G_2_/M arrest with increasing doses of HIT. In A-204, the shift towards G_0/1 _arrest caused by SAHA was comparably insignificant. The combination treatment of HIT and SAHA resulted in a slight G_2_/M shift and a total loss of cells in S-phase. In the hFOB1.19 cell line, SAHA also resulted in a slight increase of cells in G_0/1 _phase, but HIT only, as well as the combination treatment did not induce a G_0/1 _or G_2_/M arrest. In contrast, rather a decrease of cells in G_0/1 _in favor of an increased number of cells in S-phase was observed.

**Figure 9:**
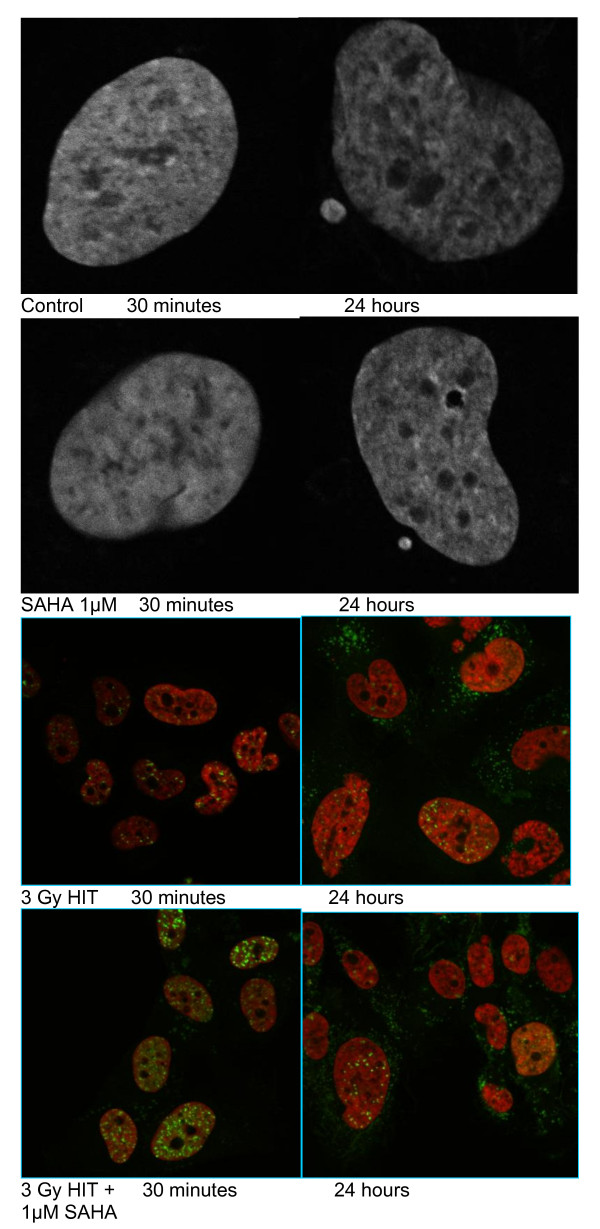
**Confocal laser scanning microscopy of KHOS-24OS**. Concocal laser scanning microscopy after staining with fluorescence markers towards chromatin (DAPI) and γH2AX. Cells were analyzed 30 min and 24 h after treatment with 1 μM SAHA, 3 Gy HIT and the combination of both.

Microscopic inspection of chromatin and γH2AX in KHOS-24OS cells after treatment with SAHA only, HIT only as well as in combination (Figure 9) revealed abundant focal accumulations of γH2AX after HIT alone as well as in combination with SAHA, especially 30 min after HIT-exposure and significantly reduced after 24 h. No significant trend-setting differences were observable after HIT only compared to combination treatment with HIT and SAHA in this cell line. Chromatin showed de-compaction 30 min after SAHA exposure as expected. This effect began to reverse within 24 h after treatment.

## Discussion

HDACI have been reported to prevent radiation-induced toxicity on normal tissue after XRT [19], while sensitizing tumor cells [7,20,21]. We are the first to show a promising, even selective effect by the combination of HIT and HDACI in infantile sarcoma cells.

We have previously reported on the ability of the HDACI SAHA to selectively radio-sensitize pediatric sarcoma cell lines to photon treatment as opposed to normal tissue cell lines [7] suggesting a therapeutic benefit by this combination. HIT is a further promising alternative to XRT in otherwise often radio-resistant sarcomas due to the superb biological effectiveness and dose conformity. However, the potential of heavy ions to cause adverse late effects such as secondary cancer must not be overlooked, especially in young and pediatric patients. Clinical studies are underway [13]. Despite the favorable clinical outcome of HIT alone, the interest in combination treatments, especially substances that ameliorate heavy ion-induced damage to normal cells, is increasing. Therefore the combination of HIT with molecularly targeted approaches like the combination with HDACI is worth investigating.

Apoptosis, necrosis, autophagy and delayed reproductive death of progeny cells are possible mechanisms for cell death after HIT. Further possible effects of HIT are cell inactivation by premature senescence as well as accelerated differentiation. Previous studies have shown that changes in chromatin organization, as induced by HDACI, modify cell morphology and de-condensate chromatin, which seems to interrelate with cellular radio-sensitivity [22]. Changes in cellular ultrastructure and increased autophagic vacuoles have been observed after heavy-ion exposure [23]. In consequence of these reports, we hoped to find further hints towards the underlying mechanism of HDACI-induced changes in sensitivity to HIT by microscopy. We observed KHOS-24OS after SAHA alone, combination treatment with HIT and SAHA and HIT only. However, no trend-setting differences in cell morphology were observable.

In contrast to our previous findings concerning the combination of XRT and SAHA in the three cell lines investigated [7], repair protein expression was not influenced by the combination of SAHA and HIT in A-204, but only in KHOS-24OS. It has previously been reported that high-LET radiation induces different changes in gene expression as compared to low-LET XRT a possible explanation for this discrepancy [24]; However, the combination of SAHA with either XRT or HIT showed similar effects on the repair kinetics of DSB as measured by γH2AX, despite the different reaction on the protein expression level immediately after treatment.

The fact that HDACI enhance radio-response to XRT of human tumor cells by impairing the repair of DNA damage has been reported earlier [22,23]. Our data show that this is also true for our infantile sarcoma cell lines and the combination with HIT. As shown by Hamada et al., γH2AX focus disappearance, i.e. DNA-repair, proved to be significantly slower after treatment with high-LET HIT than with XRT in both sarcoma cell lines, as well as the osteoblast cell line [25]. SAHA increased this effect in the sarcoma cell lines, but showed a protective effect on the osteoblast cell line. The significantly decreased number of DSB in the osteoblast cell lines after combination treatment with SAHA and HIT compared to HIT only substantiates the hope that SAHA increases the therapeutic ratio. This is consistent with our earlier results, as well as many other reports that showed the ability of HDACI to prevent radiation-induced toxicity on normal tissue after XRT [7,17],

We deliberately chose two infantile sarcoma cell lines with differing properties for our experiments. KHOS-24OS is a tetraploid osteosarcoma cell line with a known p53 mutation, A-204 a diploid and tetraploid rhabdomyosarcoma cell line with wild-type p53.

KHOS-24OS showed a lower sensitivity to XRT radiation compared to A-204 [7], which is in line to the mutated p53. Multiple pathways are involved in maintaining the genetic integrity of a cell after exposure to ionizing radiation. A common cellular response to DNA damaging agents is the activation of cell cycle checkpoints. One of the key proteins in the checkpoint pathways is the tumor suppressor gene p53, which is frequently damaged in tumor cells and mediates the two major DNA damage-dependent cellular checkpoints at the G(1)-S transition and at the G(2)-M transition [25]. p53 mutations often lead to XRT resistance [26]. While KHOS-24OS contains mutated p53, A-204 is a p53-wild type cell line [27]. High-LET irradiation treatment with heavy ions has previously been shown to be less dependent on the cellular p53 status, resulting in a lower radiobiological effectiveness (RBE) in those cells that are more sensitive to photon treatment due to p53-dependent apoptosis [28]. HDAC inhibitors are also known to enhance mechanisms leading to apoptosis independently from the p53 status [29].

The p53 mutation may explain why HIT compared to XRT has a stronger effect in KHOS-24OS than in A-204 cells. However, HIT and SAHA are promising in A-204 as well, and the synergistic effect of HIT and SAHA was altogether only a little more pronounced in the p53 mutated cell line KHOS-24OS. Radio-sensitization to HIT by SAHA thus seems to be independent of p53, as previously described for either single agent alone [30]. While p53 expression was not affected by SAHA, HIT or the combination, p21 was up-regulated in the sarcoma cell lines by SAHA as well as SAHA+HIT and down-regulated in the osteoblast cell line, correlating well to the observed apoptotic reactions of the sarcoma cell lines in contrast to hFOB1.19. p21 is known to influence cell proliferation, regulation of S-phase DNA-replication, as well as DNA-repair. The observed therapy-related changes in cell cycle progression may be induced by p21 interaction. However, in contrast to XRT, heavy ion irradiation is known to be effective at killing cells with little cell-cycle dependency [31]. While p21 does not by itself induce apoptosis, it does interact with caspase-associated, p53-independent apoptotic pathways [32].

Interestingly, however, the influence of the combination therapy of SAHA with HIT, as well as XRT on the repair kinetics as represented by the γH2AX-response, proved to be higher in A-204 than in KHOS-24OS (Figure 5). Here p53 may play a role after all. It has previously been reported, that p53 wild-type cancer cells show a faster loss of γH2AX after XRT than cells with p53 deficiency [33]. Our findings suggest that this is also true for HIT, especially when combined with SAHA. As suggested by Hamada et al., high-LET radiation may be particularly effective in patients with mutated p53 or p53 depleted tumors and the addition of a HDACI may be of additional value [25]. p21 has been demonstrated to be a predictive marker for response to HDACI treatment alone in sarcomas [34]. Our findings warrant further investigations whether p21 status of sarcomas may serve as an additional prognostic marker for the efficacy of combined HIT with HDACI.

Our study shows that SAHA is an intriguing novel adjuvant to HIT in certain sarcomas.

## Conclusion

The combination of HDACI like SAHA with HIT may be a promising strategy in the treatment of infantile sarcomas. Our data suggest an improvement of therapeutic ratio.

## List of abbreviations used

DSB: DNA double-strand breaks; HDACI: HDAC inhibitor(s); HIT: Heavy ion therapy; IF: immunofluorescence; XRT: Photon irradiation; SAHA: Suberoylanilide hydroxamic acid.

## Competing interests

The authors declare that they have no competing interests.

## Authors' contributions

SO conceived and coordinated the study, interpreted the data and drafted the manuscript, MT acquired, analyzed and interpreted the data, KR performed the microscopic analysis, KJW helped in conceiving the experiments and analyzing the data, PEH helped in conceiving the experiments and analyzing the data, RLP performed experimental procedures (gamma-H2Ax) and helped to analyze data, SB helped with all experiments that were performed with heavy ions, MB helped to interpret the data and draft the manuscript, AEK aided in study design and provided the necessary laboratory equipment, VE performed experimental procedures (FACS analysis) and helped to analyze the data, JD aided in study design and provided the necessary laboratory equipment, CB conceived and coordinated the study and interpreted the data.

All authors read and approved the final manuscript
